# The efficacy of incretin therapy in patients with type 2 diabetes undergoing hemodialysis

**DOI:** 10.1186/1758-5996-5-10

**Published:** 2013-02-28

**Authors:** Yuichi Terawaki, Takashi Nomiyama, Yuko Akehi, Hiromasa Takenoshita, Ryoko Nagaishi, Yoko Tsutsumi, Kunitaka Murase, Hisahiro Nagasako, Nobuya Hamanoue, Kaoru Sugimoto, Ayako Takada, Kenji Ito, Yasuhiro Abe, Yoshie Sasatomi, Satoru Ogahara, Hitoshi Nakashima, Takao Saito, Toshihiko Yanase

**Affiliations:** 1 Department of Endocrinology and Diabetes Mellitus, School of Medicine, Fukuoka University, 7-45-1 Nanakuma, Jonan-ku, Fukuoka 814-0180, Japan; 2 Division of Nephrology and Rheumatology, Department of Internal Medicine, Fukuoka University School of Medicine, 7-45-1 Nanakuma, Jonan-ku, Fukuoka 814-0180, Japan

**Keywords:** Type 2 diabetes, Hemodialysis, Incretin therapy, CGM, Insulin therapy

## Abstract

**Background:**

Although incretin therapy is clinically available in patients with type 2 diabetes undergoing hemodialysis, no study has yet examined whether incretin therapy is capable of maintaining glycemic control in this group of patients when switched from insulin therapy. In this study, we examined the efficacy of incretin therapy in patients with insulin-treated type 2 diabetes undergoing hemodialysis.

**Methods:**

Ten type 2 diabetic patients undergoing hemodialysis received daily 0.3 mg liraglutide, 50 mg vildagliptin, and 6.25 mg alogliptin switched from insulin therapy on both the day of hemodialysis and the non-hemodialysis day. Blood glucose level was monitored by continuous glucose monitoring. After blood glucose control by insulin, patients were treated with three types of incretin therapy in a randomized crossover manner, with continuous glucose monitoring performed for each treatment.

**Results:**

During treatment with incretin therapies, severe hyperglycemia and ketosis were not observed in any patients. Maximum blood glucose and mean blood glucose on the day of hemodialysis were significantly lower after treatment with liraglutide compared with treatment with alogliptin (*p* < 0.05), but not with vildagliptin. The standard deviation value, a marker of glucose fluctuation, on the non-hemodialysis day was significantly lower after treatment with liraglutide compared with treatment with insulin and alogliptin (*p* < 0.05), but not with vildagliptin. Furthermore, the duration of hyperglycemia was significantly shorter after treatment with liraglutide on both the hemodialysis and non-hemodialysis days compared with treatment with alogliptin (*p* < 0.05), but not with vildagliptin.

**Conclusions:**

The data presented here suggest that patients with type 2 diabetes undergoing hemodialysis and insulin therapy could be treated with incretin therapy in some cases.

## Introduction

Diabetes is a multifactorial progressive disease accompanied by subsequent systematic vascular complications. Diabetic nephropathy is one of the most critical complications for diabetic patients because it can lead to severe renal failure, which requires treatment with hemodialysis (HD). Indeed, the major cause of the need for HD is diabetic nephropathy, and this has been the case in Japan since 1998. Controlling the blood glucose level of diabetic patients on HD is difficult, because of frequent hypoglycemia, restriction of the use of anti-diabetic agents, and instability of glucose, insulin, and drug metabolites between the day of HD and the non-HD day. However, strict glycemic control is important for the prognosis of diabetic patients with or without renal impairment [1]. Thus, additional effective and tolerable medications are urgently required for diabetic patients with renal impairment.

In recent years, medications that mimic or enhance incretin activity, such as glucagon-like peptide (GLP)-1 receptor agonists and dipeptidyl peptidase (DPP)-4 inhibitors, have emerged as important new treatments for type 2 diabetes [2]. Incretins, such as GLP-1 and glucose-dependent insulinotropic polypeptide (GIP), are secreted after meals and act directly on pancreatic β cells to stimulate glucose-dependent insulin secretion. GLP-1 has multiple roles in the regulation of glucose metabolism, because it acts on pancreatic β cells and on other organs, including the brain, stomach, and vasculature [3]. However, intrinsic incretins are rapidly inactivated by DPP-4 [4], and as a result GLP-1 receptor agonists and DPP-4 inhibitors have been developed for the treatment of type 2 diabetes.

Incretin therapy is associated with additional benefits compared with other anti-diabetic agents. Firstly, incretin therapy does not induce hypoglycemia, because it controls blood glucose regulation by both insulin and glucagon secretion depending on the blood glucose level [5]. Additionally, incretin therapy may be able to protect pancreatic β cell function and volume, in contrast to sulfonylureas [6,7], and decrease blood glucose level without weight gain [8]. Recently, DPP-4 inhibitors have become one of the most frequently prescribed medications for type 2 diabetes in Japan, and we have previously reported the efficacy of a DPP-4 inhibitor, sitagliptin, in Japanese patients with type 2 diabetes [9]. Furthermore, some incretin therapies could potentially be used for blood glucose control in patients with type 2 diabetes who also have severe renal impairment [10].

According to the guidebook for chronic kidney disease (CKD) in Japan [11], DPP-4 inhibitors including 50 mg oral vildagliptin once-daily, 6.25 mg alogliptin, and the GLP-1 receptor agonist, liraglutide 0.3 mg injected once-daily, are available for treatment of patients with type 2 diabetes and end-stage renal disease (ESRD). However, there are no reports comparing the efficacy and safety of incretin therapies in diabetic patients on HD. In the present study, we examined the efficacy and safety of incretin therapy in patients with insulin-treated type 2 diabetes undergoing HD, using continuous glucose monitoring (CGM).

### Subjects and methods

#### Subjects

Ten Japanese insulin-treated type 2 diabetic patients aged 34–83 years old and on HD were recruited for this study. Patients with a history of type 1 diabetes and diabetic ketoacidosis, severe impairment of intrinsic insulin secretion (serum C-peptide <2.0 ng/dL), requirement of high dose insulin injections (≥20 U/day), severe cardiac disease (New York Heart Association grade ≥III), or severe liver disease were excluded.

Baseline characteristics of the 10 patients are shown in Table 1. In this study, we measured glycated albumin (GA) as a marker of glycemic control, because GA is a more reliable glycemic control marker than glycated hemoglobin in patients with renal failure [12]. Baseline GA was 24.1 ± 1.5% and the mean duration of diabetes was 25.4 ± 2.3 years. Duration of HD was 4.1 ± 1.1 years. Serum-C peptide (S-CPR) was 6.7 ± 1.3 ng/mL, suggesting that patients still had intrinsic insulin secretion. The total dose of daily insulin injection was 11.6 ± 1.9 U/day. No patient had the anti-glutamic acid dehydrogenase (GAD) antibody or history of ketoacidosis. During hospitalization undergoing insulin or incretin therapy, all patients were treated with HD, thrice weekly for 4–5 hours with a bicarbonate dialysate containing 100 mg/dL of glucose. The calculated single-pool Kt/V [13] was 1.44 ± 0.09, suggesting sufficient efficacy of hemodialysis.

**Table 1:** Patient characteristics at baseline

**Parameter**	**Value**
n	10
Sex (male : female)	7:3
Age (years old)	62.9±4.3
Duration of diabetes (years)	25.4 ± 2.3
Duration of HD (years)	4.1±1.1
BMI (kg/m2)	23.0±1.5
Dose of insulin (U/day)	11.6±1.9
Glycated albumin (%) *	24.1 ± 1.5
S-CPR (ng/ml)	6.7 ± 1.3
spKTV	1.44 ± 0.09
Cre (mg/dl)	9.72±0.91
PTH (pg/ml)	88.0 ± 31.4
AST (U/l)	9.3 ± 1.1
ALT (U/l)	9.7 ± 1.3
gGTP (U/l)	16.9 ± 2.2
Total choresterol (mg/dl)	162.1 ± 8.1
Triglyceride (mg/dl)	120.1 ± 20.6
Blood glucose (mg/dl)	162.1 ± 8.1
Complications	
Macroangiopathy (− / +)	(6/4)
Neuropathy (− / +)	(0/10)
Retinopathy (− / +)	(0/10)

Values are means ± SEM or n. *n=9.

All patients provided written informed consent to participate. The study protocol was approved by the ethics committees at Fukuoka University Hospital. The study was performed in accordance with the ethical principles stated in the Declaration of Helsinki, 1964, amended in Edinburgh in 2000.

## Methods

**Figure 1:**
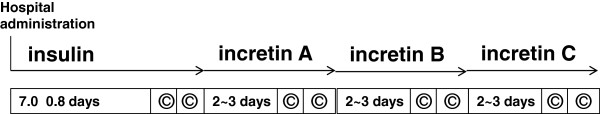
**Study protocol. **Incretin A-C indicates 0.3 mg liraglutide, 50 mg vildagliptin, or 6.25 mg alogliptin. ©: CGM monitoring day.

The present study design is shown in Figure 1. All patients were hospitalized and switched from insulin therapy to incretin therapy. After patients were admitted to hospital, blood glucose was controlled by insulin therapy targeting a fasting blood glucose level of <130 mg/dL and a 2 h post-prandial glucose level of <180 mg/dL without hypoglycemia for 7.0 ± 0.8 days (3–13 days). After insulin therapy, patients were treated with 0.3 mg liraglutide, 50 mg vildagliptin, and 6.25 mg alogliptin, in a randomized crossover design without a wash-out period.

### Continuous glucose monitoring

CGM was performed during the last 2 days of insulin therapy as a baseline evaluation, and then patients were treated with incretin therapy. After at least 2 days of treatment with 0.3 mg liraglutide, 50 mg vildagliptin, or 6.25 mg alogliptin, CGM was performed on both the day of HD and the non-HD day for each incretin therapy. CGM was performed using a CGMS System GOLD system monitor (Medtronic MiniMed Inc., Northridge, CA, USA). Changes in glucose were monitored by CGM for 2 successive days, and injection of liraglutide and administration of vildagliptin or alogliptin once-daily began at least 36 h before CGM. Based on CGM data on the last 2 days, corresponding to the day of HD or non-HD day, the maximum glucose level, minimum glucose level, average and SD of 24 hours glucose, and duration of hyperglycemia (glucose level ≥200 mg/dL) and hypoglycemia (glucose level <70 mg/dL) were determined and compared the baseline on insulin with all therapies.

### Statistical analysis

Summary statistics for continuous variables are presented as mean ± standard error. One-way analysis of variance and paired *t*-tests were performed to analyze differences between incretin therapies. A value of *p* < 0.05 was considered significant for all statistical tests.

## Results

**Figure 2:**
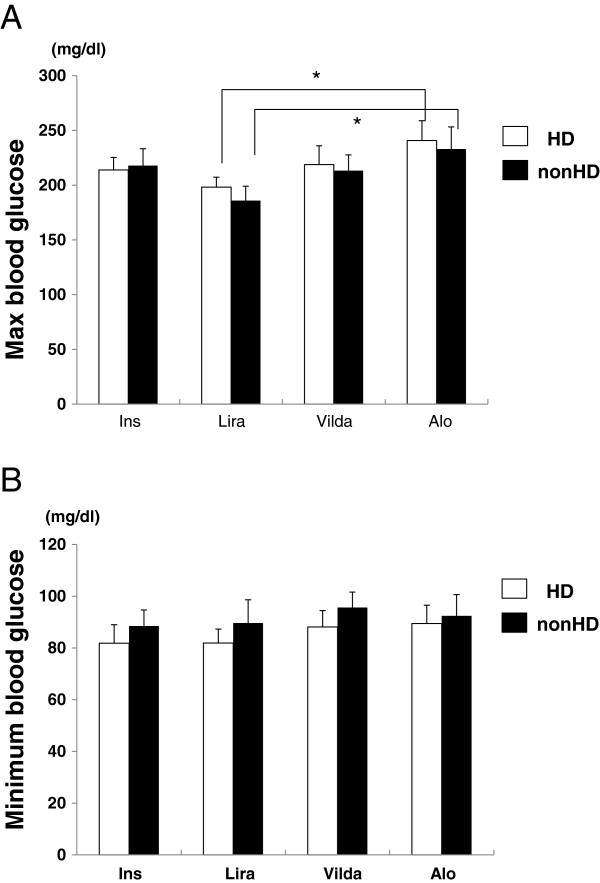
**Maximum blood glucose level (A) and minimum blood glucose level (B). **Data are presented as mean ± SEM, *p < 0.05, n = 10, Ins; insulin, Lira; once-daily injection of 0.3 mg liraglutide, Vilda; once-daily oral administration of 50 mg vildagliptin, Alo; once-daily oral administration of 6.25 mg alogliptin.

No severe hyperglycemia, ketosis, severe nausea, or other adverse effects were observed in patients at any time during incretin therapy. As shown in Figure 2A, maximum blood glucose level was approximately 200 mg/dL for all therapies (insulin; 213.9 ± 11.5 mg/dL (HD), 217.9 ± 15.5 mg/dL (non-HD), liraglutide; 198.2 ± 9.0 mg/dL (HD), 185.9 ± 131.1 mg/dL (non-HD), vildagliptin; 218.8 ± 17.1 mg/dL (HD), 213.2 ± 14.1 mg/dL (non-HD), alogliptin; 240.7 ± 18.2 mg/dL (HD), 233.0 ± 20.1 mg/dL (non-HD)). For incretin therapy, the maximum blood glucose level associated with liraglutide was significantly lower compared with treatment with alogliptin on both the day of HD and the non-HD day (*p* < 0.05), whereas there was no significant difference between liraglutide and vildagliptin. Conversely, there was no significant difference in minimum blood glucose level between the therapies (insulin; 81.8 ± 7.2 mg/dL (HD), 88.5 ± 6.2 mg/dL (non-HD), liraglutide; 81.9 ± 5.4 mg/dL (HD), 89.6 ± 9 mg/dL (non-HD), vildagliptin; 88.1 ± 6.3 mg/dL (HD), 95.6 ± 6.0 mg/dL (non-HD), alogliptin; 89.4 ± 7.1 mg/dL (HD), 92.4 ± 8.2 mg/dL (non-HD)).

We next analyzed the average and standard deviation (SD), a magnitude of glucose fluctuation, measured using CGM. As shown in Figure 2A, the average blood glucose level associated with the therapies was 120–160 mg/dL (insulin; 134.3 ± 6.7 mg/dL (HD), 148.4 ± 10.2 mg/dL (non-HD), liraglutide; 129.0 ± 6.2 mg/dL (HD), 135.9 ± 6.8 mg/dL (non-HD), vildagliptin; 144.3 ± 7.7 mg/dL (HD), 149.6 ± 7.6 mg/dL (non-HD), alogliptin; 147.4 ± 7.3 mg/dL (HD), 149.7 ± 9.1 mg/dL (non-HD)). Compared with alogliptin, liraglutide significantly decreased the average blood glucose level on the day of HD, (*p* < 0.05). Furthermore, we compared the SD of insulin and incretin therapies. As shown in Figure 2B, the SD of liraglutide was lower in comparison to other treatments (insulin; 33.4 ± 4.2 mg/dL (HD), 33.3 ± 4.7 mg/dL (non-HD), liraglutide; 27.3 ± 1.9 mg/dL (HD), 21.5 ± 1.9 mg/dL (non-HD), vildagliptin; 34.7 ± 6.1 mg/dL (HD), 30.2 ± 4.5 mg/dL (non-HD), alogliptin; 38.0 ± 6.5 mg/dL (HD), 32.0 ± 5.1 mg/dL (non-HD)). On the day of HD, the SD of liraglutide was significantly lower compared with insulin and alogliptin treatment (*p* < 0.05), but not with vildagliptin (p=0.14), suggesting that liraglutide controlled blood glucose in patients undergoing HD with smaller glucose fluctuations.

**Figure 3:**
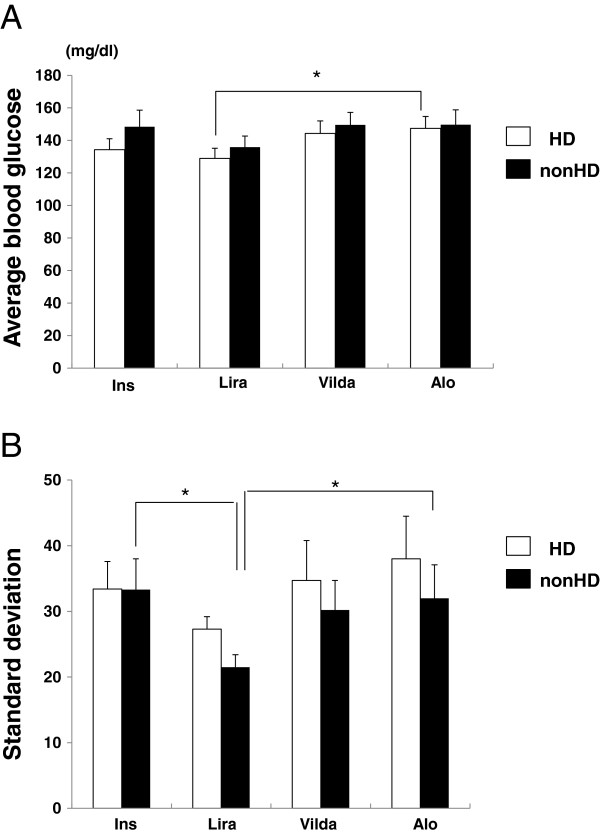
**Average glucose level (A) and standard deviation (B). **Data are presented as mean ± SEM, *p < 0.05, n = 10, Ins; insulin, Lira; once-daily injection of 0.3 mg liraglutide, Vilda; once-daily oral administration of 50 mg vildagliptin, Alo; once-daily oral administration of 6.25 mg alogliptin.

Finally, we measured hyper- (blood glucose ≥200 mg/dL) and hypo-glycemic (blood glucose <70 mg/dL) periods associated with insulin and incretin therapy. As shown in Figure 3A, liraglutide was associated with a decreased hyperglycemic period compared with other treatments (insulin; 40.0 ± 15.0 min/day (HD), 117.9 ± 42.4 min/day (non-HD), liraglutide; 22.9 ± 23.9 min/day (HD), 33.3 ± 34.4 min/day (non-HD), vildagliptin; 87.1 ± 54.6 min/day (HD), 178.7 ± 95.0 min/day (non-HD), alogliptin; 104.1 ± 38.0 min/day (HD), 77.8 ± 26.9 min/day (non-HD). Both on the day of HD and the non-HD day, the hyperglycemic period associated with liraglutide treatment was significantly shorter compared with insulin and alogliptin (*p* < 0.05), but not with vildagliptin. Conversely, there was no significant difference in the hypoglycemic period between the therapies (Figure 3B, insulin; 16.3 ± 9.6 min/day (HD), 21.7 ± 28.6 min/day (non-HD), liraglutide; 49.5 ± 70.7 min/day (HD), 23.9 ± 23.5 min/day (non-HD), vildagliptin; 1.0 ± 1.4 min/day (HD), 1.9 ± 0.0 min/day (non-HD), alogliptin; 6.8 ± 5.7 min/day (HD), 8.0 ± 7.5 min/day (non-HD)). The frequencies of hypoglycemic periods were independent from the duration of hemodialysis.

## Discussion

In the present study, all 10 type 2 diabetic patients undergoing HD were able to terminate insulin therapy permanently, and were subsequently treated with incretin therapy, including a once-daily injection of 0.3 mg liraglutide, once-daily oral 50 mg vildagliptin, and 6.25 mg alogliptin until at least 3 months after the end of this study. Blood glucose data obtained from CGM suggested that switching from insulin therapy to incretin therapy was effective and well tolerated. After CGM monitoring with incretin therapies, no patient required insulin therapy, and subsequently, six patients continued to be treated with liraglutide, two patients with vildagliptin, and two patients with alogliptin. Their GA 3 months later with incretin therapies was 22.9 ± 0.3%, which was lower compared with the baseline GA, as shown in Table 1, suggesting long term tolerability of incretin therapy in diabetic patients undergoing HD. However, this study has some limitations, because of the small sample size and short-term nature of the study. In the present study, we have shown that it may be possible to use incretin therapy in type 2 diabetes patients undergoing HD, but further study with larger sample sizes over longer terms and including multiple regression analysis of contributing factors to glycemic control by incretin therapy are required to confirm the findings here.

Blood glucose control of diabetic patients undergoing HD is difficult, because of the risk of hypoglycemia. Additionally, therapeutic options for diabetic patients with renal impairments are limited, because reduced glomerular filtration leads to an accumulation of drugs and their metabolites [14]. Before the availability of incretin therapy, standard treatment of diabetic patients with ESRD was with insulin, some glinides, or α-glucosidase inhibitors. However, incretin therapy has emerged as another option for the treatment of diabetic patients with ESRD. Incretin therapy may be an ideal treatment for patients with diabetes and ESRD, because of the low risk of hypoglycemic events. Furthermore, as previously reported by us in an animal model, incretin may also have a vasoprotective effect [15,16]. Indeed, as renal impairment is one of the risk factors that can accelerate coronary artery disease [17]. In addition, incretin has received much attention for its effects on fatty liver [18] and bone [19]. In the present study, we measured liver enzymes and parathormone (PTH) level; however, no changes were observed in these markers after incretin therapy. The lipid lowering effect, as we previously reported [9], was not observed in the present study, probably because baseline lipid level was low in the present study.

**Figure 4:**
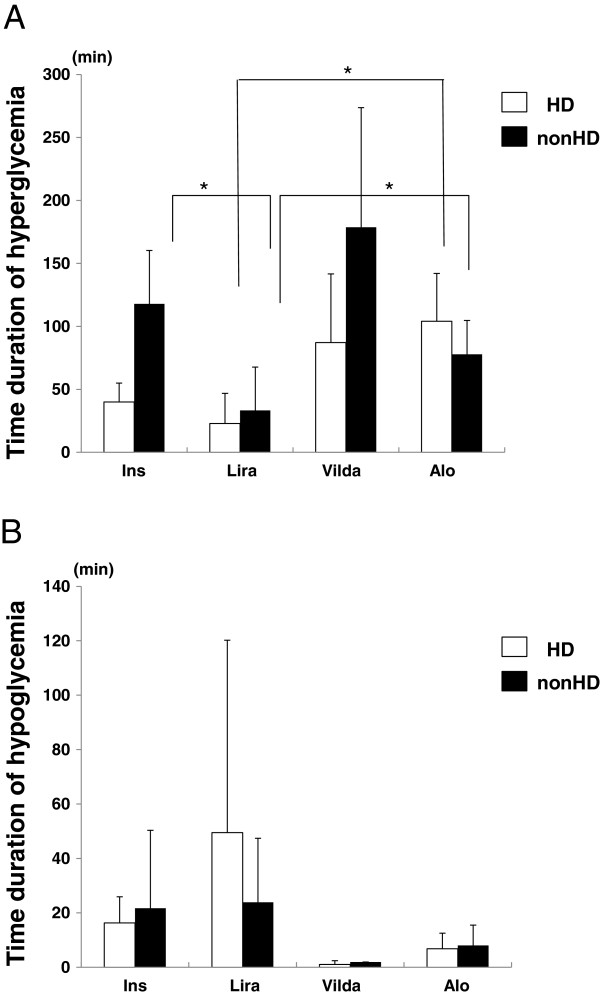
**Duration of hyperglycemia (A) and hypoglycemia (B). **Data are presented as mean ± SEM, *p < 0.05, n = 10, Ins; insulin, Lira; once-daily injection of 0.3 mg liraglutide, Vilda; once-daily oral administration of 50 mg vildagliptin, Alo; once-daily oral administration of 6.25 mg alogliptin.

In the present study, 0.3 mg liraglutide decreased blood glucose levels and fluctuations of blood glucose more compared with 50 mg vildagliptin and 6.25 mg alogliptin in diabetic patients undergoing HD. Liraglutide is a GLP-1 receptor agonist, which is available for diabetic patients with renal impairment, whereas exenatide, another GLP-1 receptor agonist, is not recommended for use by diabetic patients with severe renal impairment [20]. In a previous report, the safety and pharmacokinetics of liraglutide in subjects with varying stages of renal impairment was examined [21]. In this report, there was no significant difference in the pharmacokinetics and onset of adverse effects depending on the grade of renal impairment. However, there are no reports examining the safety of liraglutide at higher doses and over a longer term in patients with ESRD. In the present study, one patient was treated with liraglutide for over 9 months without adverse effects. However, further studies are required to confirm the safety and efficacy of liraglutide in diabetic patients undergoing HD. Theoretically, incretin therapy should not cause hypoglycemia. However, we observed hypoglycemic periods, and 0.3 mg liraglutide was associated with the highest frequency of hypoglycemia (Figure 4B). These data suggest that both hyperglycemia and hypoglycemia should be monitored when treating diabetic patients undergoing HD with incretin therapy. In the present study, the lowest hypoglycemic period, which was not significantly different from other therapies, was observed with vildagliptin.

Similar to our present study, other groups have previously demonstrated the efficacy and tolerability of the DPP-4 inhibitor, vildagliptin, in type 2 diabetic patients undergoing HD [22,23]. Kume et al. treated drug naïve type 2 diabetic patients undergoing HD with 50 mg vildagliptin once-daily for 24 weeks, and observed a significant reduction in postprandial glucose and GA from the baseline data [20]. Ito et al. treated type 2 diabetic patients undergoing HD with once-daily 50 mg or 100 mg vildagliptin for 24 weeks, and observed a significant reduction in HbA1c, GA, and postprandial glycemia [23]. Additionally, the efficacy of alogliptin in type 2 diabetic patients undergoing HD was also reported by another group [24]. Although these reports suggest efficacy and safety of DPP-4 inhibitors for the treatment of patients with type 2 diabetes undergoing HD, caution should still be exercised when treating patients undergoing HD. Because 85% vildagliptin, 76% alogliptin, and 87% sitagliptin are excreted via the kidney [25], there is a risk that these compounds may accumulate during long-term use. Very recently, linagliptin, which is primarily excreted via bile acid, has become available [26]. According to CKD guidelines [11], linagliptin does not require dose reduction even in patients with ESRD, because of the stable pharmacokinetics of this compound in such patients. Unfortunately, we were not able to include linagliptin in the present study. However, it could potentially become a treatment option for diabetic patients undergoing HD in the future.

As described above, several reports have examined the efficacy of incretin therapy in type 2 diabetic patients undergoing HD. However, there have been no reports demonstrating a switch from insulin to incretin therapy in the treatment of diabetic patients undergoing HD. In the present study, we recruited patients who had S-CPR ≥2.0 ng/dL. Because the glucose lowering effect of incretin therapy depends on intrinsic insulin secretion, S-CPR needs to be high for incretin therapy to be effective. Baseline S-CPR of patients who completed the current study was 6.7 ± 1.3 ng/mL (2.25-16.3 ng/mL), suggesting that 2.0 ng/mL might be the borderline S-CPR concentration with which insulin therapy could be switched to incretin therapy in type 2 diabetic patients undergoing HD. In addition, we recruited patients who did not require insulin injections of >20 U/day to achieve good glycemic control, based on our preliminary experience. In the present study, baseline insulin injection dose was 11.6 ± 1.9 U/day (4–19 U/day).

Furthermore, this is the first report demonstrating CGM of incretin therapy in type 2 diabetic patients undergoing HD. CGM can monitor blood glucose levels for 24 hours, and detect the average and fluctuation range of blood glucose. Because incretin therapy can decrease not only the average blood glucose, but also fluctuations of glucose level, CGM is ideal for the evaluation of glycemic control in incretin therapy [27]. In the present study, incretin therapy, especially liraglutide, controlled both the average and fluctuation of glucose level in type 2 diabetic patients undergoing HD.

## Conclusions

This study has shown that it is possible that insulin-treated type 2 diabetic patients undergoing HD might be able to switch from insulin to incretin therapy, if they have a serum C-peptide ≥2.0 ng/dL and an insulin injection dose <20 U/day. However, further studies with larger patient groups and over longer study periods are required to confirm the findings of the present study.

## Abbreviations

HD: Hemodialysis; GLP-1: Glucagon-like peptide-1; DPP-4: Dipeptidyl peptidase-4; GIP: Glucose-dependent insulinotropic polypeptide; CKD: Chronic kidney disease; CGM: Continuous glucose monitoring; GA: Glycated albumin; S-CPR: Serum-C peptide; GAD: Glutamic acid dehydrogenase; ESRD: End-stage renal disease.

## Competing interests

The authors declare that they have no competing interests.

## Authors’ contributions

YU collected data and performed statistical analyses; TN wrote the manuscript and conceived of the research hypothesis; YA, HT, RN, YT, KM, HN, NH, KS, AT, KI, YA, YS, SO, HN, and TS reviewed and edited the manuscript and assisted in patient recruitment; TN assisted in conception of the research hypothesis and reviewed and edited the manuscript. All authors read and approved the final manuscript.
